# Cloning, expression, and purification of an α-carbonic anhydrase from *Toxoplasma gondii* to unveil its kinetic parameters and anion inhibition profile.

**DOI:** 10.1080/14756366.2024.2346523

**Published:** 2024-06-07

**Authors:** Viviana De Luca, Simone Giovannuzzi, Clemente Capasso, Claudiu T. Supuran

**Affiliations:** a Department of Biology, Agriculture and Food Sciences, National Research Council (CNR), Institute of Biosciences and Bioresources, Naples, Italy; b Neurofarba Department, Pharmaceutical and Nutraceutical Section, University of Florence, Sesto Fiorentino, Italy

**Keywords:** carbonic anhydrase, anion inhibitors, Toxoplasmosis, enzyme kinetics

## Abstract

Toxoplasmosis, induced by the intracellular parasite *Toxoplasma gondii*, holds considerable implications for global health. While treatment options primarily focusing on folate pathway enzymes have notable limitations, current research endeavours concentrate on pinpointing specific metabolic pathways vital for parasite survival. Carbonic anhydrases (CAs, EC 4.2.1.1) have emerged as potential drug targets due to their role in fundamental reactions critical for various protozoan metabolic processes. Within *T. gondii*, the Carbonic Anhydrase-Related Protein (TgCA_RP) plays a pivotal role in rhoptry biogenesis. Notably, α-CA (TcCA) from another protozoan, *Trypanosoma cruzi*, exhibited considerable susceptibility to classical CA inhibitors (CAIs) such as anions, sulphonamides, thiols, and hydroxamates. Here, the recombinant DNA technology was employed to synthesise and clone the identified gene in the *T. gondii* genome, which encodes an α-CA protein (Tg_CA), with the purpose of heterologously overexpressing its corresponding protein. Tg_CA kinetic constants were determined, and its inhibition patterns explored with inorganic metal-complexing compounds, which are relevant for rational compound design. The significance of this study lies in the potential development of innovative therapeutic strategies that disrupt the vital metabolic pathways crucial for *T. gondii* survival and virulence. This research may lead to the development of targeted treatments, offering new approaches to manage toxoplasmosis.

## Introduction

Toxoplasmosis is a common parasitic infection caused by the intracellular protozoan *Toxoplasma gondii*, which belongs to the phylum Apicomplexa, a group of parasitic protozoans characterised by a unique structure known as the apical complex^1–4^. This disease is widespread globally and affects a significant portion of the human population^5^^,^^6^. *T. gondii* transmission occurs through various routes, including contact with cat faeces, ingestion of contaminated food or water, and even through vertical transmission from mother to foetus^7^^,^^8^. The complex life cycle of *T. gondii* involves multiple stages, ranging from tachyzoites responsible for acute infections to bradyzoites found within tissue cells, ultimately leading to oocyst shedding in the environment^9–12^. Surviving within its host, *T. gondii* employs various immune evasion strategies, swiftly invading cells, utilising parasitophorous vacuole membranes, and modulating host cell responses^13–16^. Despite the widespread distribution of *T. gondii*, clinical disease manifestations occur only in a limited number of individuals and animals exposed to the parasite^17^^,^^18^. The treatment landscape for toxoplasmosis remains limited, primarily relying on medications targeting enzymes involved in the folate pathway, which is critical for DNA synthesis^19–21^. Although medicines such as Spiramycin, a macrolide antibiotic, exhibit efficacy in preventing maternal-foetal transmission, treating established foetal infections remains a challenge due to their limitations in crossing the placental barrier^22–24^. Medications like Pyrimethamine (a classical antiprotozoal agent) and Trimethoprim (an antibiotic belonging to the class of dihydrofolate reductase inhibitors), often used in combination with sulphonamide antibiotics, while effective against tachyzoites, also impede DNA synthesis in healthy tissues with high metabolic activity^25^^,^^26^. Addressing the need for more effective and stage-specific treatments, research focuses on identifying crucial metabolic pathways essential for parasite survival. Numerous potential drug targets have emerged, encompassing pathways like electron transport, fatty acid synthesis, isoprenoid synthesis, calcium signalling, and gene expression control^27–31^. Among these potential targets lies a superfamily of metalloenzymes called carbonic anhydrases (CAs, EC 4.2.1.1)^32–34^. They catalyse the reversible hydration of carbon dioxide (CO_2_) to bicarbonate ions (HCO_3_^–^) and protons (H^+^)^35^. This fundamental reaction is represented as follows: CO_2_ + H_2_O ⇌ HCO_3_^–^ + H^+^ and is vital in maintaining acid-base balance, pH regulation, ion transport in different tissues and organs, and in various metabolic processes, including gluconeogenesis, urea synthesis, and fatty acid production^36^^,^^37^. In the literature, it has been described that certain CAs, like the human isoform CA VA, are associated with mitochondrial lipid synthesis through pyruvate carboxylation reactions^38–40^. In *T. gondii*, lipid metabolism holds profound significance, pivotal to its survival and pathogenicity^41^. The parasite's reliance on lipid synthesis extends beyond conventional roles as structural components to encompass multifaceted functions crucial for its life cycle within the host^42^. In 2017, Chasen et al. identified in *T. gondii* a Carbonic Anhydrase-Related Proteins (CARP), termed TgCA_RP^43^. Remarkably, TgCA_RP exhibits a close relationship with the characterised η-class CA found in *Plasmodium falciparum*^43^. TgCA_RP undergoes posttranslational modification at its C terminus, featuring a glycosylphosphatidylinositol (GPI) anchor crucial for its localisation within intracellular tachyzoites. TgCA_RP is involved in the biogenesis of rhoptries, pivotal organelles in the invasion and survival strategies of *T. gondii*. Intriguingly, the α-CA (TcCA) from another protozoan, *Trypanosoma cruzi*, was successfully isolated and studied, determining its inhibition profiles with various classes of CA inhibitors (CAIs), including anions, sulphonamides, thiols, and hydroxamates^44–46^. Among these, thiols exhibited nanomolar inhibitory activity, with some effectively inhibiting epimastigote growth in vivo in two *T. cruzi* strains^46^. These findings corroborate the hypothesis that CA from *T. gondii*, similar to TcCA, might play a role in providing bicarbonate for crucial enzymatic reactions within the protozoan. The most noteworthy discovery made by the Capasso and Supuran groups was the identification of a gene within the *T. gondii* genome that encodes a protein, whose amino acid C-terminal part resembled a CA belonging to the α-CA class^32^. In this context, the C-terminal part of the CA from *T. gondii*, referred to as Tg_CA, was synthesised, and heterologously expressed through recombinant DNA technology. Using the stopped-flow technique, the Tg_CA kinetic constants were determined. Moreover, our investigation delved into the inhibition patterns exhibited by Tg_CA when exposed to a broad spectrum of classical CAIs, known as inorganic metal-complexing compounds^47^^,^^48^. These inhibitors are appealing because of their small molecular or ionic structures and their ability to interact with the CA active site, influencing the enzyme activity^48^. This makes them potential candidates for rational design and optimisation of compounds with increased binding affinity, selectivity, and potency towards the CA encoded by parasite. Thus, this study holds substantial significance as it may contribute to the development of innovative therapeutic strategies aimed at disrupting the vital metabolic pathways crucial for the survival and virulence of *T. gondii*.

## Materials and methods

### Chemicals and instruments

The chemicals and instruments used in this study were procured from various sources. Isopropyl b-D-1-thiogalactopyranoside (IPTG) and antibiotics were purchased from Merck (Darmstadt, Germany), while the Affinity column (His-Trap FF) and molecular weight markers were obtained from Cytiva (Uppsala, Sweden). Additionally, the AKTA Prime purification system was acquired from Cytiva, the SX20 Stopped-Flow instrument from Applied Photophysics (Leatherhead, UK), and the SDS–PAGE apparatus from BioRAD (Hercules, California, USA). All remaining chemicals utilised were of reagent grade.

### Gene identification, synthesis, cloning, heterologous expression, SDS-page and protonography

The process of gene identification, synthesis, and cloning for the *T. gondii* CA (Tg_CA) gene involved several steps. Briefly, the gene encoding for Tg_CA was identified by utilising the "Protein BLAST" program^49^, employing amino acid sequences of human and bacterial α-carbonic anhydrases (α-CAs) as references. The synthetic Tg_CA gene, corresponding to the specific region (486–829) of the amino acid sequence, was custom designed by GeneArt Company (Thermo Fisher Scientific, Milan, Italy). This synthetic gene possessed specific base-pair sequences (CACC) at its 5′ end, crucial for directional cloning in the pMK-T vector (a subcloning vector from Thermo Fisher Scientific, Milan, Italy). Subsequently, the Tg_CA gene was cloned into the expression vector pET100/D-TOPO (Invitrogen, Palo Alto, CA, USA), yielding the plasmid pET100D-Topo/Tg_CA. To ensure the integrity of the gene and absence of errors at the ligation sites, the vector containing the fragment underwent bidirectional automated sequencing. The pET100D-Topo/Tg_CA vector was employed to transform competent Escherichia coli BL21 (DE3) codon plus cells (Agilent)^50^. The induced cellular culture with IPTG resulted in the overexpression of the recombinant Tg_CA. Post-growth (3 h), the cells were harvested and disrupted via sonication. Tg_CA was produced as a His-tag fusion protein with a 6xHis tag at the N-terminal of the polypeptide chain. Subsequently, the cellular extract was purified using a nickel affinity column (His-Trap FF) connected to an AKTA Prime system. The elution of the recombinant Tg_CA from the column was achieved using an elution buffer composed of specific concentrations of Tris, imidazole, and sodium chloride^50^. The recovered Tg_CA exhibited a purity of 90%. Protein quantification was performed using the Bradford method by BioRAD^51^. For analysis, a 12% Sodium Dodecyl Sulphate-polyacrylamide gel electrophoresis (SDS-PAGE) was employed, following which Coomassie Brilliant Blue-R staining was conducted^52^. Additionally, protonography was performed on the SDS-PAGE gel to detect yellow bands indicating hydratase activity, following the method outlined by Capasso and colleagues^53^. The CA activity assay was carried out based on the conversion of CO_2_ to bicarbonate, monitored by pH variation and utilising bromothymol blue as an indicator^54^. This assay was conducted at 0 °C, and Wilbur-Anderson units were calculated to determine enzyme activity^54^.

### Phylogenetic analysis

Multialignment of amino acid sequences was performed using the program MUSCLE 3.1 (MUltiple Sequence Comparison by Log-Expectation), a new computer program for creating multiple alignments of protein sequence^55^. The dendrogram was constructed using the program PhyML 3.0 searching for the tree with the highest probability^56^.

### Structural sequence alignment and Tg_CA model

The utilisation of Swiss Model, a fully automated protein structure homology-modelling server, represents a sophisticated approach in computational biology for predicting protein structures based on homologous templates^57^. Accessible via the Expasy web server, Swiss Model streamlines the process of protein structure prediction, particularly through homology modelling. In the context of modelling Tg_CA, the procedure involves several steps. Firstly, the target sequence of Tg_CA is inputted into the Swiss Model server. Subsequently, the server employs a comprehensive template library to identify suitable structural templates that share homology with the target sequence. These templates are proteins whose structures are experimentally determined and are evolutionarily related to the target protein. Once the templates are identified, the next step involves structural sequence alignment. This process aligns the target sequence with the identified templates, ensuring that regions of similarity and divergence are appropriately matched. This alignment is crucial as it serves as the basis for generating the homology model. After the alignment, the Swiss Model server employs algorithms and techniques to generate a three-dimensional model of the target protein, Tg_CA, based on the aligned template structures. The resulting model provides insights into the putative structure and conformation of Tg_CA.

### Exploring the rates of enzymatic reaction catalysed by Tg_CA

An Applied Photophysics stopped-flow instrument was used to examine the kinetic parameters of the Tg_CA catalysed reaction of carbon dioxide CO_2_ hydration^58^. To assess this activity, phenol red, present at a concentration of 0.2 millimolar (mM), was utilised as an indicator. The instrument operated at the absorbance peak of 557 nanometres (nm). The experimental conditions included a buffer solution of 20 mM Hepes adjusted to a pH of 7.5 and 20 mM NaClO_4_ to maintain a constant ionic strength (for hCA II) or NaCl (at the same concentration) in the case of Tg_CA measurements, since perchlorate showed a weak inhibition against this enzyme which could lead to errors for the measurements of the kinetic and inhibition parameters. The study observed the initial rates of the CA-catalysed CO_2_ hydration reaction within a timeframe of 10 to 100 s. The range of CO_2_ concentrations tested spanned from 1.7 mM to 17 mM to determine the kinetic parameters using Lineweaver–Burk plots and to assess inhibition constants^59^. For each inhibitor, a minimum of six data traces, specifically from the initial 5–10% of the reaction, were utilised to determine the initial velocity. The rates of the uncatalyzed reaction were determined in a similar manner and subtracted from the overall observed rates to determine the CA-catalysed reaction rates. The inhibitors were initially prepared as stock solutions ranging from 10 mM to 100 mM in distilled-deionized water. Subsequently, dilutions were made up to 0.01 mM using the assay buffer. The inhibitor and enzyme ­solutions were combined and pre-incubated for 15 min at room temperature before the assay, allowing for the formation of the enzyme-inhibitor complex or any potential hydrolysis of the inhibitor mediated by the enzyme's active site. The inhibition constants were calculated using non-linear least-squares methods employing PRISM 3 and the Cheng-Prusoff equation^60^, consistent with prior reports^61^. These values represent the average derived from a minimum of three separate determinations. The other CAs used in this study were recombinant and produced in-house. The salts and small molecules utilised were of the highest purity commercially available and sourced from Merk (Darmstadt, Germany).

## Results and discussion

### Unravelling sequence, structural features, evolutionary relationship, and catalytic proficiency of Toxoplasma gondii α-CA

**Figure 1.**
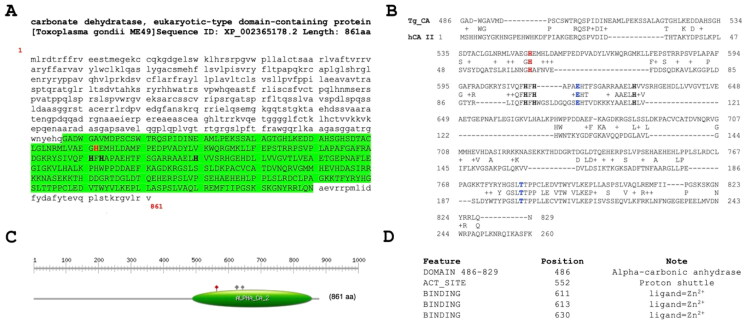
Sequence Analysis of T. gondii α-Carbonate Dehydratase. (A and C) PROSITE Motif Identification. The C-terminal region of the *T. gondii* enzyme exhibits a conserved motif characteristic of α-CA enzymes (highlighted in green). (B) Sequence alignment. Sequence alignment of Tg_CA with the hCA II reveals conserved residues typical of α-CA enzymes in the *T. gondii* protein. Distinctive short and long amino acid insertions were observed in the *T. gondii* enzyme compared with their human counterparts. (D) α-CA features. Comprehensive display of the α-CA domain and conserved residues in the *T. gondii* enzyme, including the proton shuttle and the histidines involved in the Zn^2+^ coordination. Legend: proton shuttle (highlighted in red bold); histidines of the catalytic pocket (highlighted in black bold); specific amino acids that regulate access to the active site of the enzyme (highlighted in blue bold).

By analysing the *T. gondii* genome using bioinformatic tools^49^, we identified a gene encoding 861 amino acid residues with the following ID: XP_002365178.2 and named carbonate dehydratase, eukaryotic-type domain-containing protein, archetypal strains *T. gondii* ME49 (Please visit the following link for details regarding the gene TGME49_259950: TGME49_259950 Gene Details). Utilising PROSITE^62^, a database that catalogues protein families, domains, and functional sites through patterns and profiles, a typical motifs characterising an α-CA class was identified in its C-terminal region (Figure 1(a,c)).

Subsequent alignment with hCA II revealed a comprehensive display of conserved residues characteristic of α-CA enzymes (see Figure 1(b,d)). These residues notably encompass the catalytic triad, gatekeeper and proton shuttle amino acids involved in catalytic reactions, providing a compelling insight into the primary structure and the possible functional characteristics of the identified enzyme. Consequently, the identified enzyme exhibits considerable potential to significantly contribute to bicarbonate provision, thereby highlighting its pivotal role as a key participant in the metabolic processes critical for the survival and proper functioning of *T. gondii* physiological functions. The described scenario is notably intriguing due to the findings reported by Chasen et al. (2017) regarding the *T. gondii* genome^43^. Specifically, Chasen and colleagues identified a gene within this genome responsible for encoding a carbonic anhydrase-related protein (TgCA_RP, TGME49_297070 gene). TgCA_RP was found to be inactive, as it exhibited a substitution involving two out of the three histidines within the canonical catalytic triad, characteristic of α-carbonic anhydrases (α-CAs). Similar to the η-CA, the third histidine was occupied by a glutamine, while the first histidine was a phenylalanine. These alterations occurred within a domain crucial for Zn^2+^ binding, explaining the total loss of the activity for TgCA_RP. The authors also noted a similar situation across other apicomplexan orthologs^43^.

**Figure 2.**
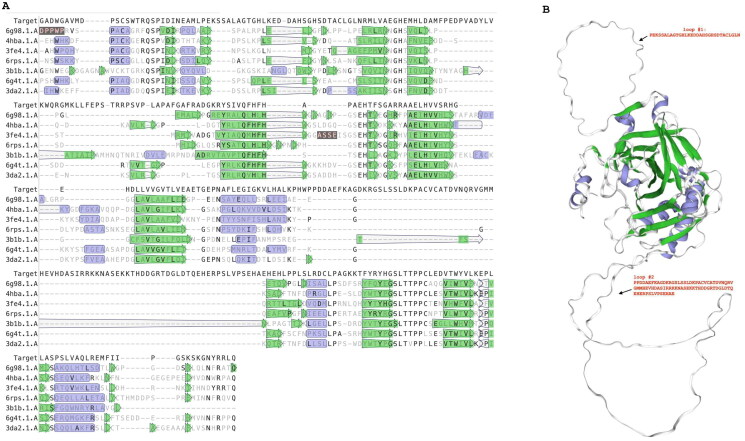
Structural sequence alignment and Tg_CA model. Left side: Using the SWISS-MODEL template library, the structural templates were identified and aligned with the target sequence of Tg_CA. Right side: ribbon representation of the overall fold of the obtained Tg_CA model (region 486–489, see Figure 1(A)) superimposed with the hCA VI structure. Legend: Target, Tg_CA model; 6g98.1.A., hCA IX in complex with sulphonamide; 4hba.1.A, Thermal and Acid Stable Variant of hCA II; 3fe4.1.A., hCA VI; hCA XII complexed with a theranostic monoclonal antibody fragment; 3b1b.1.A, alpha-CA1 from *Chlamydomonas reinhardtii*; 6g4t.1.A, hCA VII; hCA XIII. Violet indicates secondary structure colour for alpha helices; green indicates secondary structure colour for beta strands. Loops reported in the Tg_CA model (right site) are indicated by #1 and #2 with their respective amino acid sequences.

The examination of sequence alignment presented in Figure 1(B) unveiled a distinctive feature characterising the *T. gondii* α-CA, marked by short and long insertions of amino acid residues absent in the human CA counterpart (hCA II). This discovery prompted us towards a deeper investigation of *T. gondii* enzyme. Using the SWISS-MODEL template library^57^, which is a large structural database of experimentally determined protein structures derived from the Protein Data Bank, structural templates of Tg_CA were identified and aligned with the target sequence (Tg_CA) (Figure 2(A)). The structural alignment, depicted in Figure 2(A), validated the presence of the Tg_CA insertions. From this alignment, a model of Tg_CA was built and superimposed with the human CA VI structure (one of the structures selected to build the Tg_CA model) (Figure 2(B)). It is evident that Tg_CA exhibits the fundamental architectural characteristics of a classical α-CA, featuring a central β-sheet flanked by helical connections, and possessing a conical cavity as the putative active site, extending from the protein surface to the core of the molecule. The most important difference arising from the superposition is the presence of two loops formed by 31 and 82 amino acid residues, respectively (Refer to the top and bottom on the right side of Figure 2) in contrast to its human counterpart. The discovery of these distinctive loops not only adds an element of mystery to the structural landscape of Tg_CA but also calls for further investigation.

**Figure 3.**
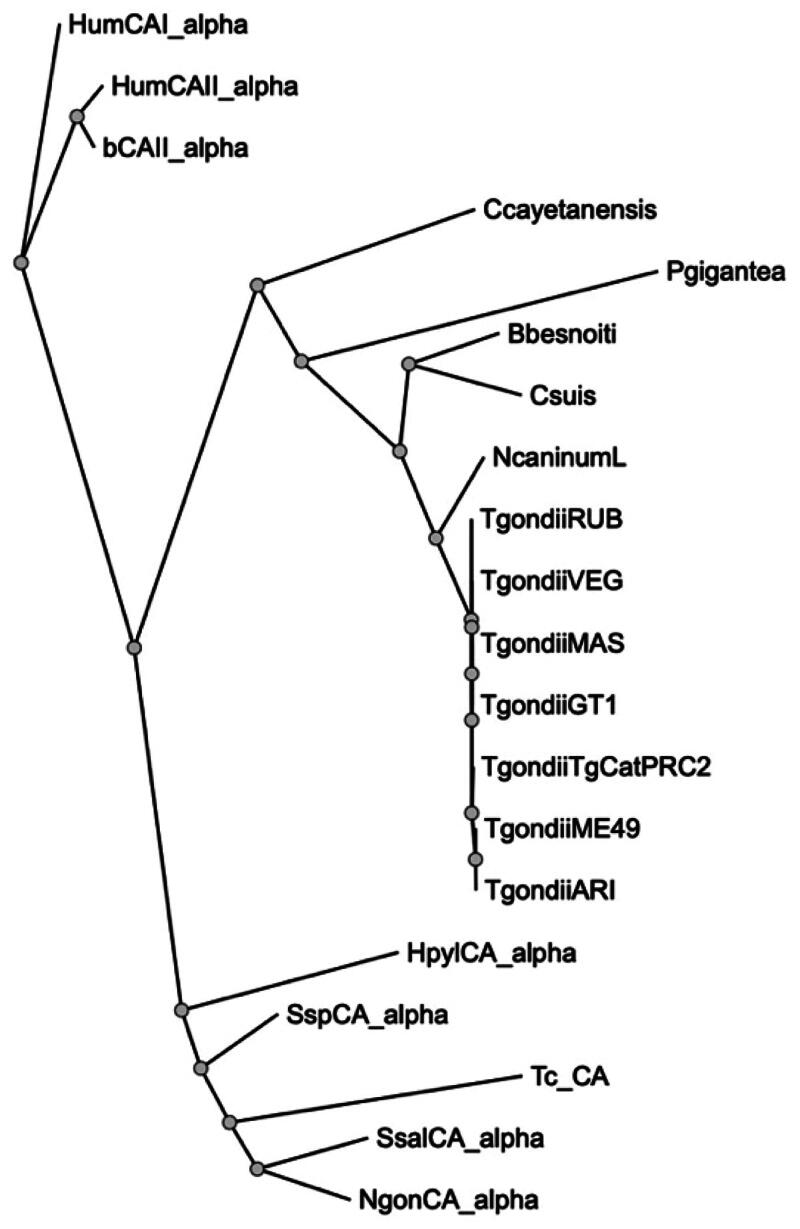
Phylogenetic analysis. A dendrogram was obtained using PhyML to generate a bootstrap consensus tree (100 replicates). The analysis utilised the following amino acid sequences: **HumCA1_alpha**, *Homo sapiens* CA I isoform, Sequence ID: NP_001122301.1; **HumCAII_alpha**, *Homo sapiens* CA II, Sequence ID: AAH11949.1; **bCAII_alpha**, *Bos taurus* CA II, Sequence ID: NP_848667.1; **Ccayetanensis**, *Cyclospora cayetanensis* alpha CA, (ID: XP_022592761.2); **Pgigantea**, *Porospora cf. gigantea* A hypothetical protein KVP18_002643 (ID: KAH0475491.1); **Bbesnoiti**, *Besnoitia besnoiti* carbonate dehydratase, eukaryotic-type domain-containing protein (ID: XP_029221989.1); **Csuis**, *Cystoisospora suis* carbonate dehydratase, eukaryotic-type domain-containing protein (ID: PHJ22557.1); **NcaninumL**, *Neospora caninum* Liverpool carbonate dehydratase, eukaryotic-type domain containing protein (ID: CEL66890.1); **TgondiiRUB**, *Toxoplasma gondii* RUB carbonate dehydratase, eukaryotic-type domain-containing protein (ID: KFG65258.1); **TgondiiVEG**, *Toxoplasma gondii* VEG carbonate dehydratase, eukaryotic-type domain-containing protein (ID: ESS32017.1); **TgondiiMAS**, *Toxoplasma gondii* MAS carbonate dehydratase, eukaryotic-type domain-containing protein (ID: KFH15199.1); **TgondiiCT1**, *Toxoplasma gondii* GT1 carbonate dehydratase, eukaryotic-type domain-containing protein (ID: EPR62766); **TgondiiTgCatPRC2**, *Toxoplasma gondii* TgCatPRC2 carbonate dehydratase, eukaryotic-type domain-containing protein (ID: KYK70381.1); **TgondiiME49**, *Toxoplasma gondii* Tg_CA (ID: XP_002365178.2); **TgondiiARI**, *Toxoplasma gondii* ARI carbonate dehydratase, eukaryotic-type domain-containing protein (ID: KYF41591.1); **HpylCA_alpha**, *Helicobacter pylori* α-CA (ID: WP_010882609.1); **SspCA_alpha**, *Sulfurihydrogenibium* sp. YO3AOP1 α-CA (ID: WP_012459296.1); **Tc_CA**, *Trypanosoma cruzi* α-CA (ID: XP_806287.1); **SsalCA_alpha**, *Streptococcus salivarius* PS4 α-CA (ID: EIC81445.1); **NgonCA_alpha**, *Neisseria gonorrhoeae* α-CA (ID: WP_003688976.1).

Thus, we performed a comprehensive phylogenetic analysis to investigate the evolutionary relationships between the protozoan Tg_CA and various eukaryotic α-CA amino acid sequences^56^. Our study encompassed an array of enzymes, including two human isoform enzymes (hCA I and II), bovine and bacterial enzymes, α-CAs identified in other protozoa belonging to the phylum Apicomplexa, and the α-CA derived from the protozoan *Trypanosoma cruzi*. The resulting dendrogram, visualised in Figure 3, notably delineates a distinct clustering pattern. It is evident that all α-CAs identified within the archetypal strains of *T. gondii* form an exclusive cluster since they were well separated from α-CAs found in other protozoa within the same phylum. Particularly noteworthy is the positioning of the *T. cruzi* α-CA (TcCA) within the dendrogram; it clusters alongside bacterial enzymes rather than associating closely with the CA enzymes found in organisms belonging to the phylum Apicomplexa. This intriguing clustering pattern strongly suggests a closer evolutionary affinity between TcCA and bacterial enzymes rather than with the α-CA counterparts from the Apicomplexa phylum.

**Figure 4.**
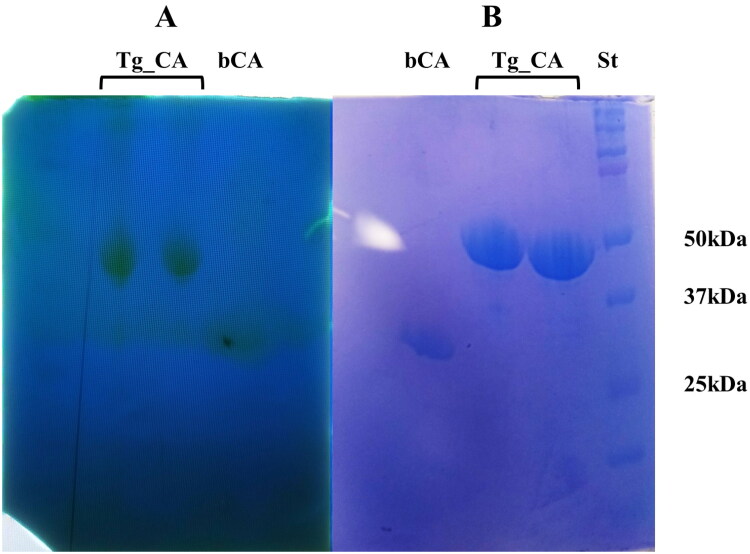
Protonography and SDS-PAGE of recombinant Tg_CA. Affinity chromatography purified Tg_CA was mixed with Loading Solution Buffer (LSB) containing SDS 0.1% and loaded onto the gel in duplicate. (a) Developed protonogram showing the CO_2_ hydratase activity of Tg_CA. (b) SDS-PAGE of purified recombinant Tg_CA. The yellow bands (lane Tg_CA, panel A) on the protonogram correspond to the enzyme activity responsible for the drop in pH from 8.2 to the transition point of the dye in the control buffer. SDS-PAGE demonstrated that these bands had an apparent molecular weight of 47 kDa (lane Tg_CA, panel B). The bCA lane (panel A and B) contains the commercial bovine CA used as a positive control, while the molecular markers are shown in lane St.

Considering the distinctive structural and evolutionary characteristics identified within the *T. gondii* α-CA, a strategic approach was undertaken to produce the recombinant Tg_CA. To facilitate purification and subsequent analysis, the recombinant Tg_CA was engineered as a fusion protein, incorporating a tail consisting of six histidine residues, commonly referred to as a His-Tag, positioned at the N-terminal end. The heterologous overexpression was initiated by inducing the *E. coli* BL21 DE3 Codon plus cells with IPTG. To facilitate proper protein folding, a supplement of 0.5 mM ZnCl_2_ was introduced to the host cells. This specific concentration of ZnCl_2_ aimed to support the correct folding of the protein. Notably, a significant portion of the intracellular recombinant protein was successfully retrieved from the soluble bacterial cellular extract following a preparation process involving sonication and subsequent centrifugation. Employing an affinity column (His-select HF Nickel affinity gel) facilitated the purification of the Tg_CA fusion protein to a state of homogeneity, as demonstrated by analysis through SDS-PAGE, showing as a subunit with an apparent molecular weight of about 47 kDa (Figure 4(B)). The purified His-tagged Tg_CA underwent comprehensive investigation to assess its catalytic activity. Our laboratories employed a specialised technique termed protonography^53^^,^^63^^,^^64^, conducted on a polyacrylamide gel, to examine and evaluate the catalytic capabilities of the purified protein (Figure 4(A)). This innovative approach allowed for the detailed analysis and visualisation of the enzyme's catalytic function directly on the gel. The resulting protonogram depicted in Figure 4(A) revealed a discernible yellow band attributed to the production of ions (H^+^) during the CO_2_ hydration reaction. The protonogram provided clear evidence that the produced recombinant Tg_CA was active and exclusively present in the monomeric state upon treatment with LSB (Laemmli Sample Buffer).

### Kinetic parameters and anion inhibition profile of Tg_CA and their significance

Lipids serve as integral components of *T. gondii* cellular architecture, contributing to the formation and maintenance of membranes and organelles^65^. Beyond structural relevance, lipids assume vital roles as signalling molecules and mediators of host-parasite interactions^65^. They actively participate in intercellular signalling, modulating host immune responses, and potentially influencing the parasitic manipulation of host cell functions. By engaging in these signalling processes, lipids play a pivotal role in the parasite's ability to evade host defences and ensure its persistence within the host cells. Moreover, lipid metabolism serves as a dynamic source of energy for *T. gondii*. The efficient utilisation and storage of lipids provide essential reserves, crucial for the parasite's adaptation during different stages of its life cycle, aiding its survival under varying environmental conditions within the host^66–68^. Lipid metabolism holds paramount importance for parasites, serving diverse functions from structural components to signalling molecules and energy stores. CAs are known to be involved not only in pH regulation but also in metabolism and signalling^56^^,^^63^.Thus, by studying the α-CA (TGME49_259950) encoded by the *T. gondii* genome holds significant importance within the context of parasite's survival strategies. Thus, we investigated the His-tagged Tg_CA catalytic activity and inhibition profiles with small molecule and anion inhibitors, known to interact with other CAs^64^.The kinetic characterisation of Tg_CA has been performed for the physiologic, CO_2_ hydrase reaction^52^, using stopped flow experiments (Table 1), and by comparing the obtained parameters with those of other α-CAs from mammals (human isoforms hCA I and II) or protozoans, TcCA from *T. cruzi*^40^.

**Table 1.** CO_2_ hydration reaction kinetic parameters of α-CA isozymes hCA I and II (of human origin), and protozoan such enzyme: TcCA from *Trypanosoma cruzi* and Tg_CA from *T. gondii*, (at 20 °C and pH 7.5) and acetazolamide (5-acetamido-1,3,4-thiadiazole-2-sulphonamide) inhibition data.

Enzyme	k_cat_	K_m_	k_cat_/K_M_	K_I_(acetazolamide)
	(s^−1^)	(mM)	(M^−1^ x s^−1^)	(nM)
Tg_CA	2.16 x 10^5^	16.0	1.34 x 10^7^	45.7
hCA I^a^	2.00 x 10^5^	4.0	5.00 x 10^7^	250
hCA II^a^	1.40 x 10^6^	9.3	1.50 x 10^8^	12
TcCA ^b^	1.21 x 10^6^	8.1	1.49 x 10^8^	61.6

^a^ Human recombinant isozymes, stopped flow CO_2_ hydrase assay method, from ref.^34^^,^^45^

^b^ Recombinant enzyme, stopped flow CO_2_ hydrase assay method, this work.^40^

The kinetic data of Table 1 show that Tg_CA possesses a medium catalytic activity for the hydration of carbon dioxide to bicarbonate, with a k_cat_ of 2.16 x10^5^ s^−1^, a K_M_ of 16.0 mM and k_cat_/K_M_ of 1.34x 10^7^ M^−1^x s^−1^, parameters which are quite similar to those of hCA I, a highly abundant red blood cell enzyme^34^. Tg_CA is roughly ten times less active compared to hCA II, a catalytically highly efficient isoform or TcCA, which shows kinetic parameters similar to those of hCA II^40^. However, although less efficient as a catalyst of the CO_2_ hydration reaction compared to hCA II or TcCA, Tg_CA shows anyhow a significant such activity, and furthermore, this activity is inhibited by the classical sulphonamide inhibitor in clinical use acetazolamide, with a K_I_ of 45.7 nM (Table 1), which is a general CAI of all classes of CAs investigated so far^45^^,^^63^^,^^64^.Indeed, only hCA II has a higher affinity for acetazolamide (K_I_ of 12 nM) whereas TcCA and hCA I show inhibition constants of 61.6–250 nM. Even so, this sulphonamide CAI is widely used clinically for the management of various diseases^34^.

A large number of inorganic/organic anions and small molecules showing CA inhibitory activity45 were assayed as inhibitors of Tg_CA (Table 2) and the obtained data were compared to those for the inhibition of the physiologically most relevant host enzyme, i.e. hCA II. The following should be noted regarding inhibition data of Table 2:

**Table 2.** Inhibition of human isoforms hCA II and the protozoan enzyme from *Toxoplasma gondii* α-CA (Tg_CA) with anions, by a CO_2_ hydrase, stopped-flow assay^52^.

Inhibitor	K_I_ (mM)^a^	
	hCA II	Tg_CAα
F^–^	>300	>100
Cl^–^	200	>100
Br^–^	63	>100
I^–^	26	>100
CNO^–^	0.03	49.6
SCN^–^	1.6	31.9
CN^–^	0.02	23.1
N_3_^–^	1.5	>100
HCO_3_^–^	85	>100
CO_3_^2–^	73	98.2
NO_3_^–^	35	8.5
NO_2_^–^	63	6.4
HS^–^	0.04	45.7
HSO_3_^–^	89	9.1
SO_4_^2–^	>200	6.0
SnO_3_^2–^	0.83	6.9
SeO_4_^2–^	112	11.1
TeO_4_^2–^	0.92	2.5
P_2_O_7_^4–^	48.5	35.7
V_2_O_7_^4–^	0.57	39.8
B_4_O_7_^2–^	0.95	63.9
ReO_4_^–^	0.75	3.4
RuO_4_^–^	0.69	2.3
S_2_O_8_^2–^	0.084	26.6
SeCN^–^	0.086	>100
CS_3_^2–^	0.0088	4.8
Et_2_NCS_2_^–^	3.1	3.1
ClO_4_^–^	>200	77.8
BF_4_^–^	>200	25.0
FSO_3_^–^	0.46	2.7
PF_6_^–^	>100	1.9
CF_3_SO_3_^–^	Nt	7.1
NH(SO_3_)_2_^2–^	0.76	8.5
H_2_NSO_2_NH_2_	1.13	0.92
H_2_NSO_3_H	0.39	0.68
Ph-B(OH)_2_	23.1	5.1
Ph-AsO_3_H_2_	49.2	9.1

^a^ Mean from 3 different assays, by a stopped-flow technique (errors were in the range of ± 5–10% of the reported values). Nt = not tested.

- All halides, azide, bicarbonate, and selenocyanate did not inhibit Tg_CA up to 100 mM, although some of them are rather effective hCA II inhibitors (e.g. selenocyanate shows a K_I_ of 0.086 mM for hCA II);
- Carbonate, tetraborate and perchlorate were poor inhibitors of Tg_CA, with K_I_s in the range of 63.9–98.2 mM. The perchlorate case is again of note, since this anion is generally not at all inhibitory against other CAs, with K_I_s > 200 mM. This is also the reason why chloride was used in the assay system of Tg_CA kinetic and inhibition assay, instead of perchlorate generally used to measure these parameters for other CAs^45^^,^^52^.
- Cyanate, thiocyanate, cyanide, hydrogensulfide, diphosphate, divanadate, peroxydisulfate and tetrafluoroborate were more inhibitory compared to the previously mentioned anions, with K_I_s in the range of 23.1–49.6 mM (Table 2). Again some non-expected behaviour may be notes, since the highly metal-complexing anions (cyanate, thiocyanate, cyanide, hydrogensulfide) showing a potent binding to hCA II; were quite poor inhibitors of Tg_CA, whereas tetrafluoroborate, which is not inhibitory against many CAs, including hCA II; showed a rather effective 25 mM inhibition constant against the protozoan enzyme.
- Effective anion inhibitors were nitrate, nitrite, bisulphite, sulphate, stannite, selenite, tellurate, perrhenate, perruthenate, trithiocarbonate, *N*,*N*-diethyldithiocarbamate, fluorosulfonate, haxafluorophosphate, triflate, iminodisulfonate, phenylboronic acid and phenylarsonic acid, with K_I_s ranging between 1.9 and 11.1 mM (Table 2). Again, important differences of inhibitory profiles are seen between the human and protozoan enzyme (e.g. haxafluorophosphate is not inhibitory against hCA II and is a quite effective Tg_CA inhibitor; trithiocarbonate is a low micromolar hCA II inhibitor, being several orders of magnitude less effective against Tg_CA, etc.).
- The most effective anion/small molecule Tg_CA inhibitors were sulfamide and sulphamic acid (sulfamate) with K_I_s ranging between 0.68–0.92 mM. It has been shown earlier by kinetic and crystallographic data that they directly bind to the zinc ion within the CA II active site^65^ ,similar to the main class of organic CAIs, the sulphonamides (and of course their isosteres, the organic sulfamides/sulfamates)^45^.

## Conclusions

The Tg_CA kinetic parameters and inhibition constants determined here for the first time provide a crucial foundation for advancing research, guiding hypotheses, and potentially paving the way for future therapeutic interventions against *T. gondii* infections. We observe that similar to other α_CAs, such as the human slow isoform hCA I, Tg_CA has a medium catalytic activity for the CO2 hydration reaction, when compared to that of highly active such catalysts, e.g. hCA II or TcCA. However, this is a highly significant data, since the k_cat_/K_M_ of 1.34x 10^7^ M^−1^x s^−1^ of Tg_CA means that more than10 millions CO_2_ molecules are being converted each second to bicarbonate, by an enzyme molecule. This is probably crucial for the pathogen, both for regulating pH and for metabolic or CO_2_ sensing processes^32^, which are very much involved in the life cycle, virulence and pathogenesis mechanisms of *T. gondii*. Developing more effective antiprotozoal drugs is quite challenging^69–72^, due to the unknown phenomenon related to the various phase cycles of such pathogens and the difficulty to grow some of them in laboratory cultures for developing new drugs. Thus, *in vitro* investigations as the one reported here may be helpful for facilitating and speeding up such processes. Some of the small molecules/anion inhibitors investigated here showed indeed promising inhibitory activity against Tg_CA (e.g. sulfamide, sulphamic acid, trithiocarbonate, *N,N*-diethyldithiocarbamate, etc.) and are amenable to facile derivatizations that may lead to the development of more effective CAIs. This study highlights significant differences in inhibition profiles between Tg_CA and the human CA II enzyme. This divergence could have implications for the development of specific inhibitors targeting *T. gondii* while minimising off-target effects on human CA enzymes. Work is in progress in these laboratories for finding effective, nanomolar such compounds with potential utility for developing new antiprotozoal agents.

## Data Availability

We will provide access to the data upon readers' request.
